# Supplementary material for: End Sequence Analysis Toolkit (ESAT) expands the extractable information from single-cell RNA-seq data
Source: Genome Res. 2016 Oct;26(10):1397–410. doi: 10.1101/gr.207902.116 (PMC5052061; doi:10.1101/gr.207902.116)
Supplement: Supplemental Material [file supp_gr.207902.116_Supp_Materials.tar.gz › ESAT-esat_v0.1/doc/html/classbroad_1_1pda_1_1samtools_1_1_paired_end_t_d_f.html]

ESAT: broad.pda.samtools.PairedEndTDF Class Reference


ESAT


- Main Page
- Related Pages
- Packages
- Classes
- Files

- Class List
- Class Index
- Class Hierarchy
- Class Members

All Classes Namespaces Files Functions Variables Enumerator Pages

- broad
- pda
- samtools
- PairedEndTDF

Public Member Functions |
Static Public Member Functions |
Public Attributes |
Protected Member Functions |
List of all members

broad.pda.samtools.PairedEndTDF Class Reference

Inheritance diagram for broad.pda.samtools.PairedEndTDF:

[legend]

Collaboration diagram for broad.pda.samtools.PairedEndTDF:

[legend]

|  |  |
| --- | --- |
| Public Member Functions | |
| void | extendPairedEndReads (final File inSamOrBam, final File outSamOrBam) throws Exception |
|  | |

|  |  |
| --- | --- |
| Static Public Member Functions | |
| static void | main (final String[] args) |
|  | |

|  |  |
| --- | --- |
| Public Attributes | |
| String | USAGE |
|  | |
| File | INPUT |
|  | |
| File | OUTPUT |
|  | |
| int | WINDOW = 25 |
|  | |
| String | GENOME = "mm9" |
|  | |
| boolean | KEEP\_INTERMEDIATE = false |
|  | |
| boolean | INCLUDE\_DUPLICATES = false |
|  | |
| int | MAX\_INSERT = 2000 |
|  | |

|  |  |
| --- | --- |
| Protected Member Functions | |
| int | doWork () |
|  | |
| String[] | customCommandLineValidation () |
|  | |

## Detailed Description

Author
:   engreitz September 9, 2012

## Member Function Documentation

|  |  |  |  |  |
| --- | --- | --- | --- | --- |
| String [] broad.pda.samtools.PairedEndTDF.customCommandLineValidation | ( |  | ) |  |

protected

|  |  |  |  |  |
| --- | --- | --- | --- | --- |
| int broad.pda.samtools.PairedEndTDF.doWork | ( |  | ) |  |

protected

Here is the call graph for this function:

|  |  |  |  |
| --- | --- | --- | --- |
| void broad.pda.samtools.PairedEndTDF.extendPairedEndReads | ( | final File | *inSamOrBam*, |
|  |  | final File | *outSamOrBam* |
|  | ) |  | throws Exception |

Here is the caller graph for this function:

|  |  |  |  |  |  |
| --- | --- | --- | --- | --- | --- |
| static void broad.pda.samtools.PairedEndTDF.main | ( | final String[] | *args* | ) |  |

static

Stock main method.

Parameters
:   |  |  |
    | --- | --- |
    | args | main arguments |

## Member Data Documentation

String broad.pda.samtools.PairedEndTDF.GENOME = "mm9"

boolean broad.pda.samtools.PairedEndTDF.INCLUDE\_DUPLICATES = false

File broad.pda.samtools.PairedEndTDF.INPUT

boolean broad.pda.samtools.PairedEndTDF.KEEP\_INTERMEDIATE = false

int broad.pda.samtools.PairedEndTDF.MAX\_INSERT = 2000

File broad.pda.samtools.PairedEndTDF.OUTPUT

String broad.pda.samtools.PairedEndTDF.USAGE

**Initial value:**

=

"Converts a paired-end SAM or BAM file to a TDF file for IGV,\ncounting coverage by fragments instead of individual reads.\n"

int broad.pda.samtools.PairedEndTDF.WINDOW = 25

---

The documentation for this class was generated from the following file:

- java/broad/pda/samtools/PairedEndTDF.java


---

Generated on Fri Aug 15 2014 15:11:01 for ESAT by  

 1.8.7
